# Supplementary material for: Cell shape-independent FtsZ dynamics in synthetically remodeled bacterial cells
Source: Nat Commun. 2018 Oct 18;9:4323. doi: 10.1038/s41467-018-06887-7 (PMC6193997; doi:10.1038/s41467-018-06887-7)
Supplement: Supplementary file 1 — Supplementary Information [file 41467_2018_6887_MOESM1_ESM.pdf]

Supplementary Information for

Cell shape-independent FtsZ dynamics in synthetically remodeled bacterial cells

Söderström et al.

Supplementary Note 1

Giant blobs produce both short and long FtsZ filaments

We found that by allowing drug-treated *E. coli* cells to grow on flat agarose pads for an extended period of time (> 10 h), cells produced internalized chains of FtsZ-mNeonGreen clusters of varying length, similar to what was observed in the shaped cells (Supplementary Figure 5). Each chain contained an average of four bundled clusters, with individual clusters measuring on average 130.5 ± 28.6 nm long and 80.2 ± 4 nm wide (n = 137), which corresponds well with previously measured dimensions for FtsZ clusters in untreated cells [1]. In some extreme cases, FtsZ-mNeonGreen chains spanned the entire cell diameter as what appeared to be one long cable of bundled filaments, while in other cells, FtsZ-mNeonGreen appeared as intricate networks similar to those of tubulin in eukaryotes (Supplementary Figure 6). Similar observations have previously been made for purified FtsZ in lipid vesicles [2]. However, in contrast to the lipid vesicles, no detectable shrinkage was observed in the cells described in this study. Furthermore, no movement of FtsZ bundles was observed in these large cells, unlike what has been reported for purified FtsZ and FtsA on supported membranes *in vitro* [3].

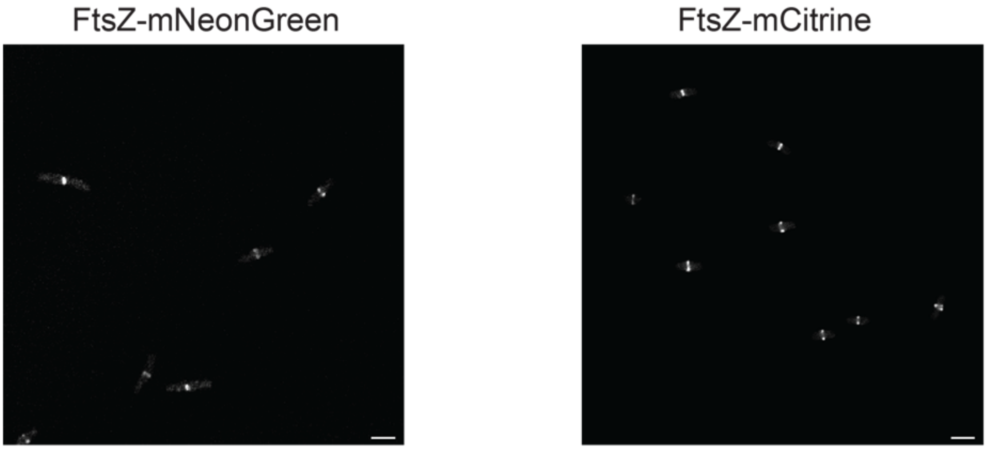

Supplementary Figure 1. Cells expressing either FtsZ-mNeonGreen or FtsZ-mCitrine produce normal-looking Z-rings.

E. coli cells with FtsZ-mNeonGreen as the only cellular source of FtsZ were grown at 30 °C in M9 minimal media supplemented with 0.2% glucose, 0.1 % casamino acids and 2 μg ml^{-1} thiamine. Plasmid-expressed FtsZ-mCitrine was induced with 2.5 μM IPTG in the presence of native FtsZ (in WT strain MC4100) at 37 °C in LB. Under these conditions, both strains produced sharp-looking Z-rings comparable to strains producing FtsZ-GFP. Scale bars = 2 μm.

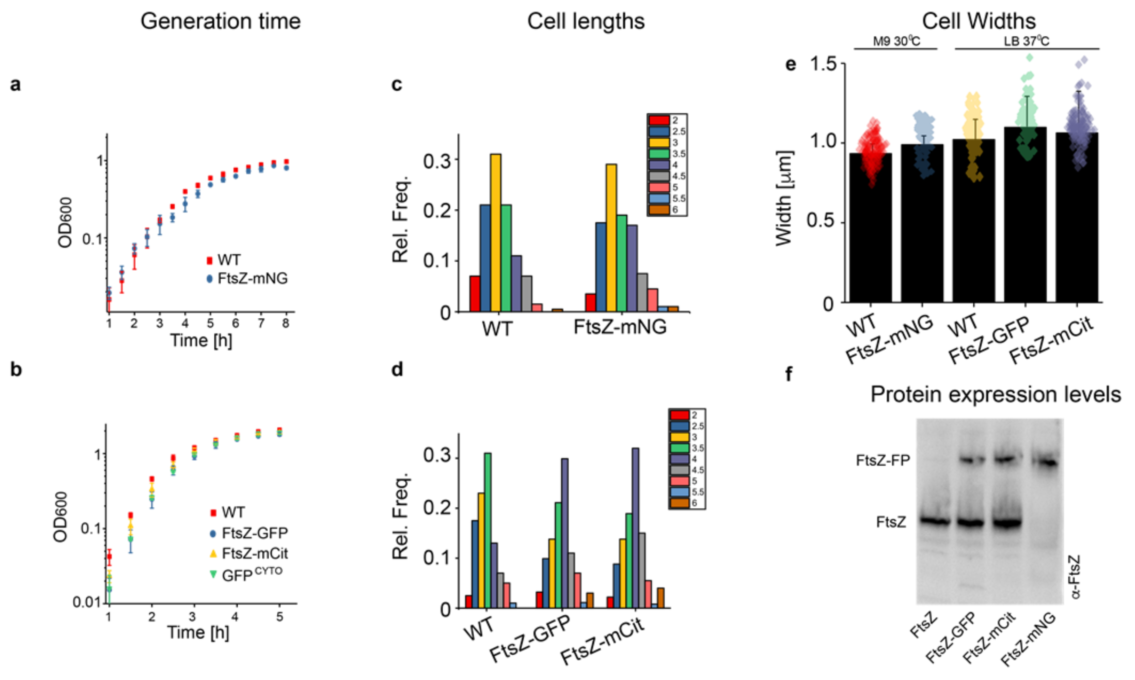

Supplementary Figure 2. Cell growth of strains expressing FtsZ-FPs.

E. coli cells expressing FtsZ-mNeonGreen (blue circles) were grown in (**a**) M9 minimal media (supplemented with 0.2% glucose, 0.1 % casamino acids and 2 µg ml^{-1} thiamine) at 30 °C, while cells expressing FtsZ-GFP (blue circles), FtsZ-mCitrine (yellow triangles) or GFP^{CYTO} (green triangles) were grown in (**b**) rich media LB at 37 °C (supplemented with 25 µg ml^{-1} ampicillin). Relative frequency of cell lengths (**c** and **d**) and widths (**e**) of strains expressing fluorescently-tagged FtsZ were similar to those of a WT stain (MC4100) (red squares). Growth curves, n = 3. At least 120 cells ($n_{WT\_M9\_30C}$ = 144, $n_{FtsZ\text{-}mNG\_M9\_30C}$ = 121, $n_{WT\_LB\_37C}$ = 122, $n_{FtsZ\text{-}GFP\_LB\_37C}$ = 120, $n_{FtsZ\text{-}mCit\_LB\_37C}$ = 139) from each strain were included in statistical analyses of length and width. Colored dots represent individual data points. (**f**) Protein expression levels of FtsZ-GFP and FtsZ-mCitrine were 27 ± 9 and 30 ± 12 % (mean ± S.D.) of total cellular FtsZ levels, respectively. Minimal protein degradation was observed in all strains expressing fluorescent proteins. n = 3. Error bars represent S.D.

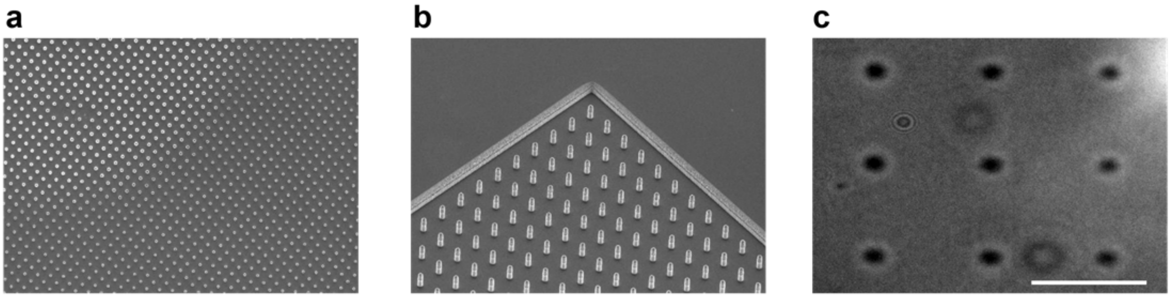

Supplementary Figure 3. Examples of round micron pillars in arrays.

The micron pillars were produced as described previously[1] (see material and methods for details). Round pillars were engineered to have widths between 1 and 3.5 μm in order to allow the cells to fit. Heights of the pillars were between 4.5 and 6 μm. Representative SEM images of the micron pillar arrays are shown. **a**, Overview image of a micron pillar array. **b**, Close-up of a corner of an array at 45 degrees side view. **c**, Brightfield image of a holey pattern produced in an agarose pad (5 % w/v in water) in which the cells were trapped. Scale bar = 5 μm.

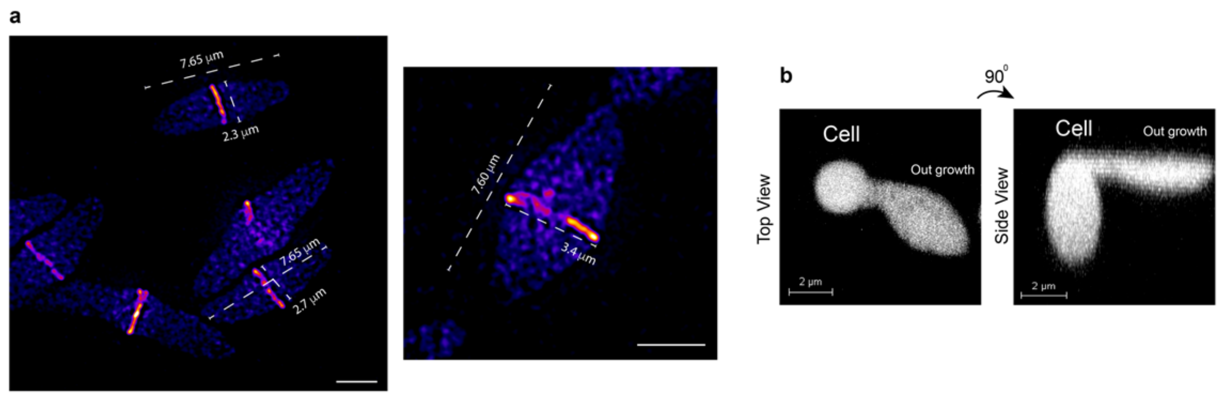

Supplementary Figure 4. Effect of A22 and cephalexin treatment on *E. coli* cells.

a, *E. coli* cells expressing FtsZ-GFP exposed to 16 µM A22 for 10 minutes were placed on an agarose pad (4 % [w/v] agarose in M9 supplemented with 40 µM A22 and 20 µg ml^{-1} cephalexin) and imaged after an additional 30 minutes. **b**, *E. coli* cell expressing cytoplasmic GFP in a micron-sized hole in an agarose pad (4 % [w/v] agarose in M9 supplemented with 40 µM A22 and 20 µg ml^{-1} cephalexin). The cell outgrew the hole and continued to grow on the agarose pad. Scale bars = 2 µm.

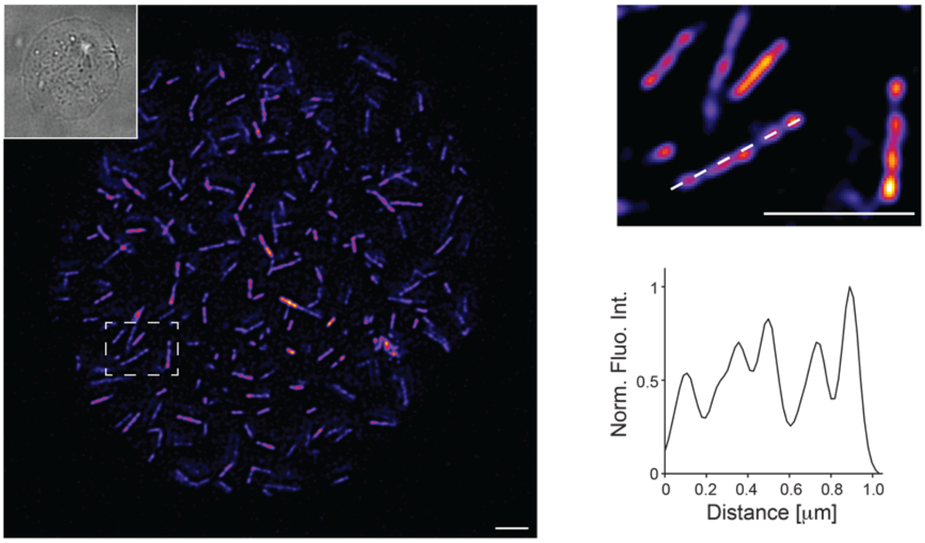

Supplementary Figure 5. FtsZ bundles in giant cells.

E. coli cells treated with drugs after an extended period of time (> 10 h) gave rise to "mega cells". In the majority of these cells, chains of FtsZ-mNeonGreen bundles formed. The bundles were on average 130.5 ± 28.6 nm long and 80.2 ± 4 nm wide (mean ± S.D., n = 137), organized in chains with an average of four bundles each. Inset shows corresponding brightfield image. Striped box is enlarged to the right, showing typical FtsZ-mNeonGreen bundles. Graph below shows the normalized fluorescence intensity profile along the dotted line. Scale bars = 1 μm.

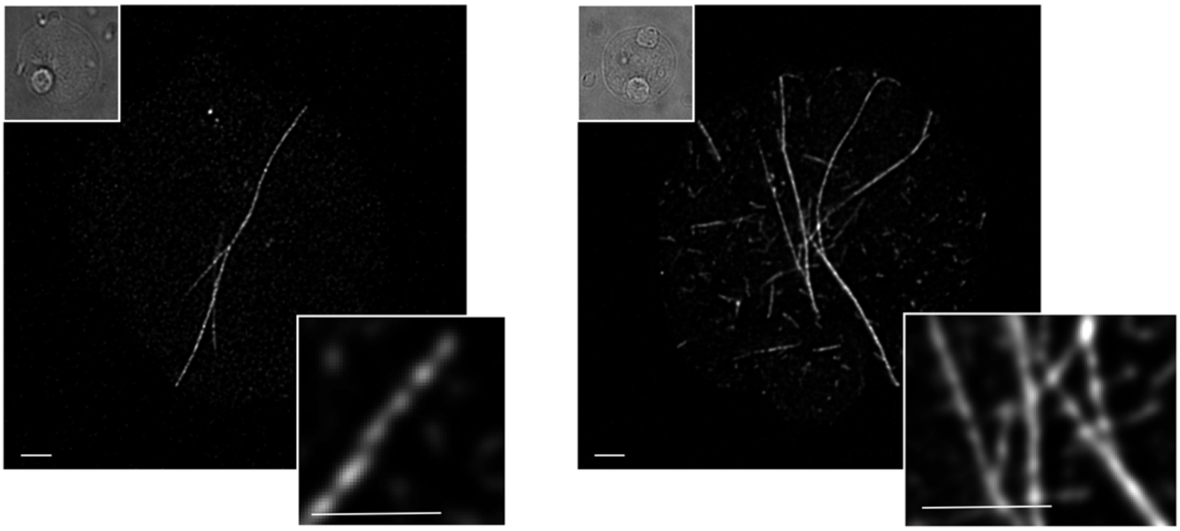

Supplementary Figure 6. Extreme FtsZ bundles in giant cells.

Drug-treated *E. coli* cells sometimes contained cell-spanning chains of FtsZ-mNeonGreen bundles that formed after an extended period of time (> 10 h). These bundles often formed what appeared to be intricate networks similar to tubulin networks in eukaryotic cells. Insets upper left show corresponding brightfield images. Insets lower right show representative close-ups of selected sections of longer chains. Scale bars = 1 μm.

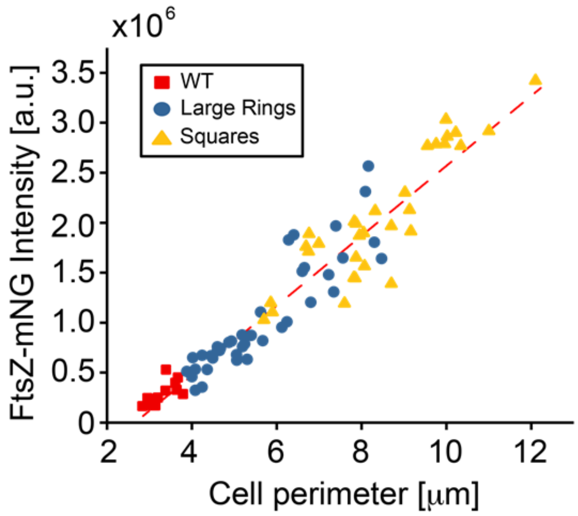

Supplementary Figure 7. FtsZ-mNeonGreen fluorescence intensity levels in various cell sizes and shapes.

The total (ring + cytoplasmic) FtsZ-mNeonGreen fluorescence intensity at the division plane in cells trapped in a standing position was found to increase with increasing cell perimeter, pointing to a possible upregulation of FtsZ expression as cells grow larger. Red squares indicate WT cells, blue circles indicate cells with large rings, while yellow triangles represent square shaped cells. The red dashed line is a fit of the data (y = -914650+351849x, R^2 =0.91553). Note that FtsZ-mNeonGreen was the only cellular source of FtsZ in these cells. n = 86.

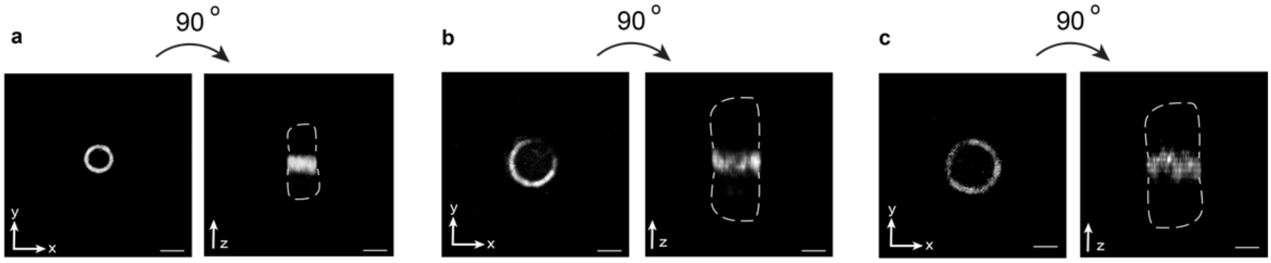

Supplementary Figure 8. Cells trapped in wells have only one Z-ring.

E. coli cells expressing FtsZ-mNeonGreen and trapped in holes were imaged using confocal Z-stacks to ensure that only one Z-ring was present per cell. **a**, An untreated cell seen from above (left) and from the side (right). **b** and **c**, Drug-treated cells with larger diameters seen from above (left) and from the side (right). Note that the z axis is elongated. Scale bars = 1 μm.

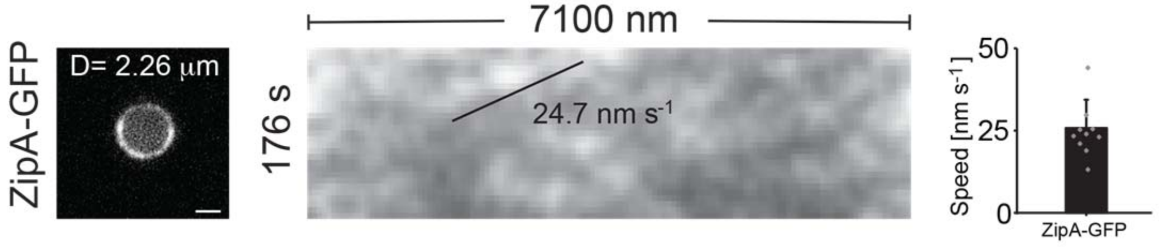

Supplementary Figure 9. ZipA-GFP in drug-treated cells moves as in untreated cells.

E. coli cells expressing ZipA-GFP exposed to A22 and cephalexin. Large cells trapped in micron-sized holes were subjected to time-lapse imaging. Left; Snapshot image from a time-lapse sequence. Scale bar = 1 μm. Middle; kymograph generated from the time-lapse sequence. Right; Average speed of ZipA-GFP in cells with diameter between 2 and 3 μm. Grey dots represent individual data points, bar represents mean with error bars representing S.D. n = 10.

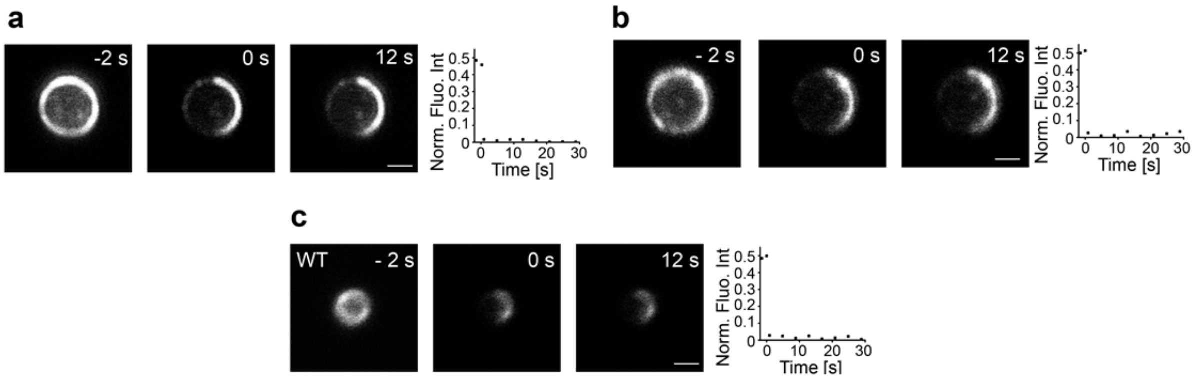

Supplementary Figure 10. Cells are stationary when trapped vertically in micron-sized holes.

FRAP measurements on fixed cells (expressing FtsZ-GFP) trapped in a vertical position showed minimal rotational movement. **a** and **b**, Drug-treated cells with large radii. **c**, WT cell. Images show typical snapshot images from FRAP measurements, from left to right; 2 seconds prior to bleaching (-2 s), momentarily after bleaching (0 s) and 12 seconds post-bleaching (12 s). Scale bars = 1 μm. Graphs show normalized fluorescence intensity of the bleached area over time.

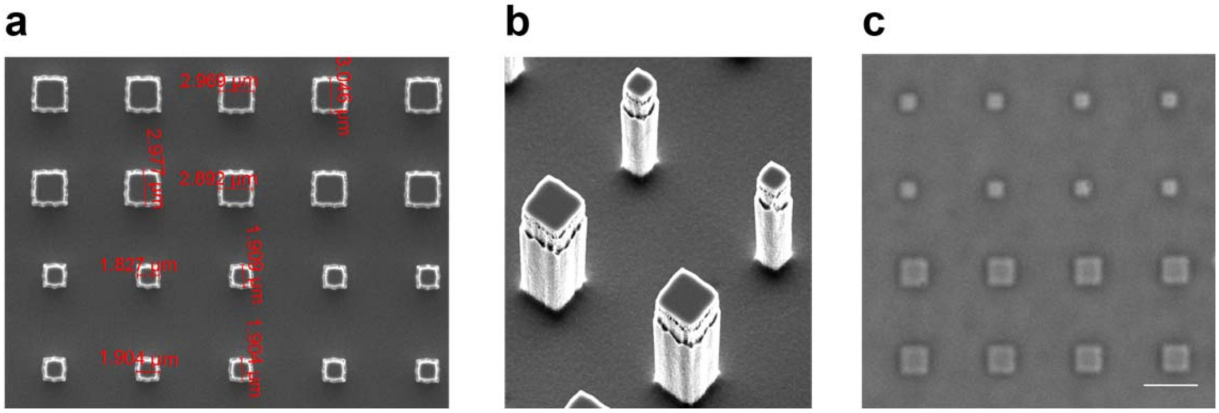

Supplementary Figure 11. Example images of arrays of square micron-sized pillars.

Micron pillars were produced as described previously [1] (see material and methods for details). Example SEM images of the micron pillar arrays are shown. **a**, Overview image of square micron pillars of part of an array. Square pillars were engineered to have side lengths between 1.8 and 3.5 μm in order to allow the cells to fit. Pillar height was set to 5.5 ± 0.5 μm. **b**, Close-up of an array at 45 degrees side view. **c**, Example of micron-sized square holes in an agarose pad. Scale bar = 5 μm.

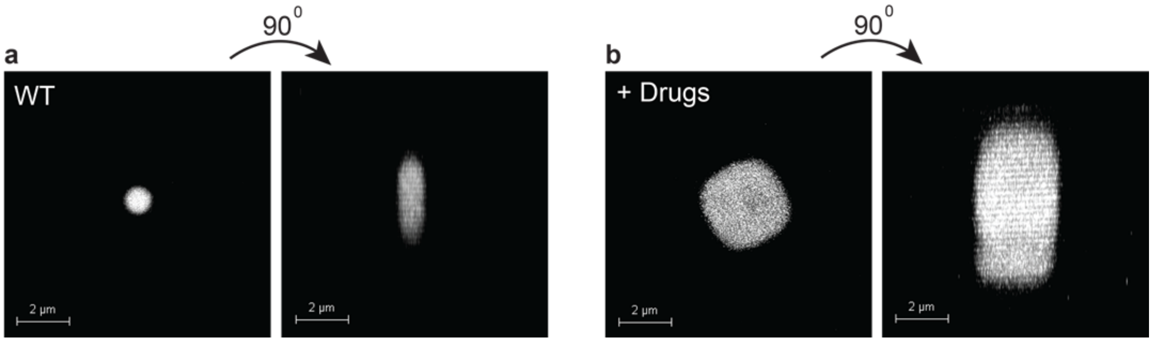

Supplementary Figure 12. Size difference between untreated and drug-treated cells.

3D renderings from confocal Z-stacks acquired on *E. coli* cells trapped standing in micron holes. **a**, Untreated cell expressing cytoplasmic GFP, visualized from above (left) and from the side (right). A gross estimation of the volume ($V_{cylinder} = h * \pi r^2$) of this particular cell is ~ 2.1 μm^3. **b**, A22 and cephalexin-treated cell expressing cytoplasmic GFP sculpted in a square hole, visualized from above (left) and from the side (right). A gross estimation of the volume ($V_{rectangular\ cuboid} = h * a^2$) of this particular cell is ~ 36 μm^3, roughly 17 times larger than the untreated cell in **a**. Note that the untreated cell is not entirely newborn and has grown in some length. Scale bars = 2 µm.

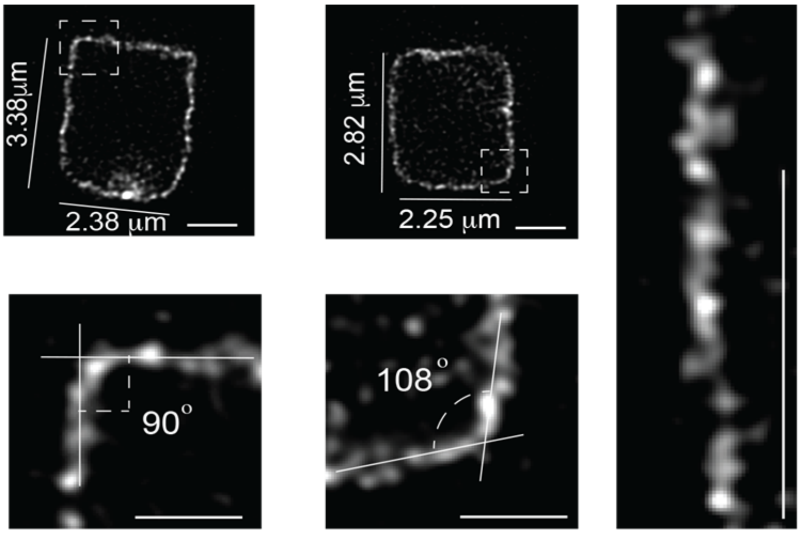

Supplementary Figure 13. Representative cells expressing FtsZ-mNeonGreen in rectangular-shaped micron holes.

Examples of drug-exposed *E. coli* cells expressing FtsZ-mNeonGreen and shaped as rectangles, imaged by STED. Close-ups of dashed regions are shown below, with angle sizes indicated. Scale bars = 1 μm.

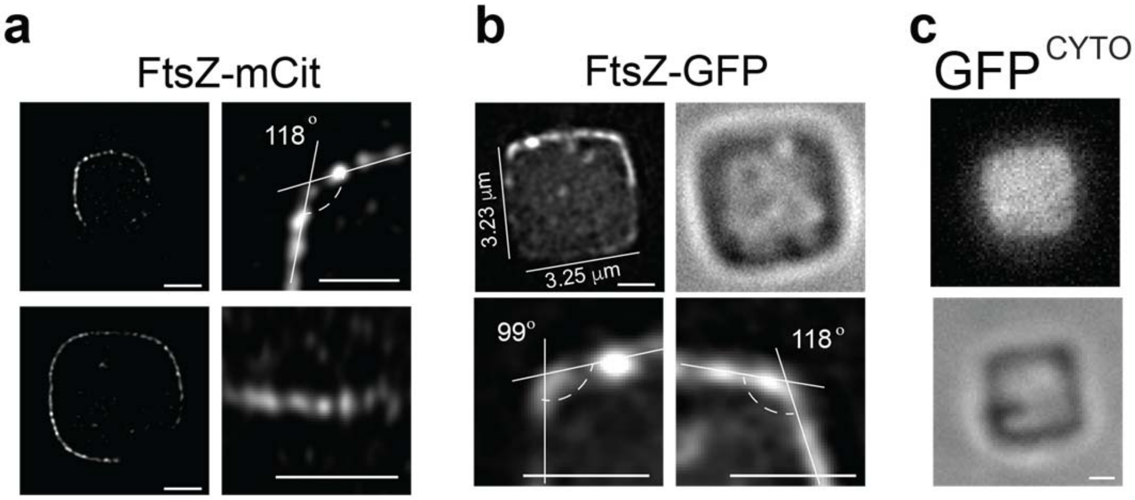

Supplementary Figure 14. Cells expressing FtsZ-mCitrine or FtsZ-GFP adapt to rectangular shapes when trapped in rectangular micron holes.

Representative images of cells expressing **a**, FtsZ-mCitrine imaged by STED or **b**, FtsZ-GFP imaged by SIM. Both strains were treated with drugs and reshaped in square micron holes. FtsZ clusters can be observed in both sharp corners and in flat membrane segments. **b**, Upper right corner shows corresponding brightfield image. **c**, Representative square-shaped cell expressing cytosolic GFP. Upper, GFP. Lower, brightfield. Scale bars = 1 μm.

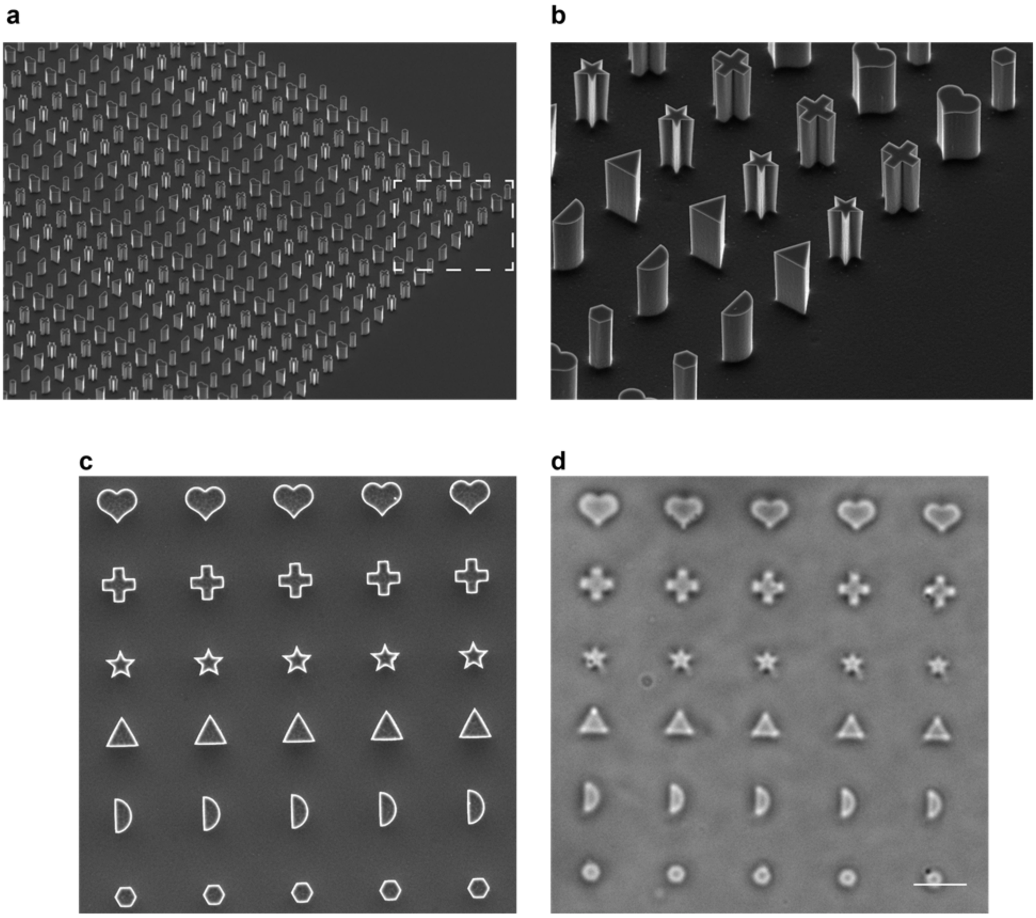

Supplementary Figure 15. Examples of micron pillar arrays with different shapes.

a - **c**, Example SEM images of multiple micron pillars. **a**, Overview image of micron pillar arrays consisting of different shapes, with pillar heights set to 5.4 μm. **b**, Close-up of the white box in **a**. Image seen from 45 degrees side view. **c**, Micron pillar structures viewed from above. **d**, Example of different-shaped micron-sized holes in an agarose pad, viewed from above. Scale bar = 5 μm.

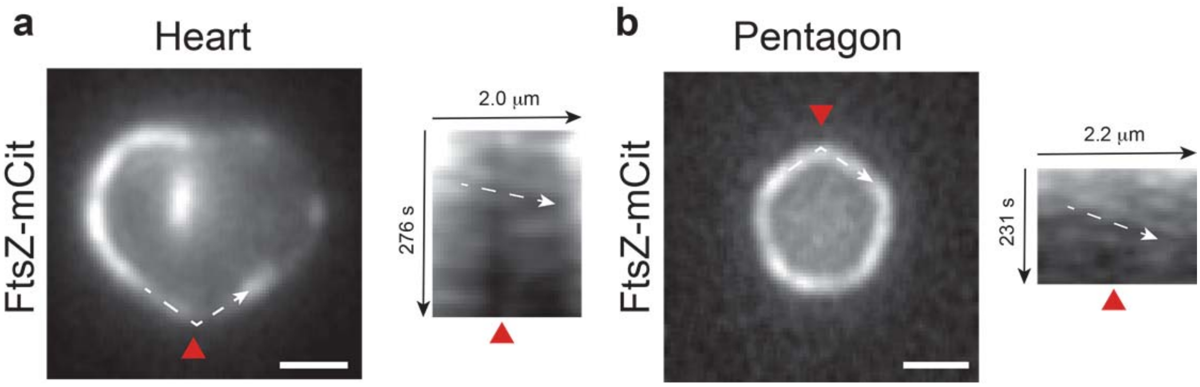

Supplementary Figure 16. FtsZ clusters treadmill continuously over sharp angles in the membrane geometry of shaped cells.

Images from time-lapse series of shaped cells (**a**) Heart and (**b**) Pentagon. Next to each image is the corresponding kymograph from the dashed line in the images. Dashed white arrows in the kymographs are placed next to representative FtsZ traces. Extreme points in the cell geometry are indicated by red arrow heads in the images and in the corresponding kymographs. The average treadmilling speed over 27 sharp-angled regions was 28.7 ± 11.1 nm s^{-1} (mean ± S.D.). We found no apparent correlation between cell shape and treadmilling speed. Scale bars = 1 μm.

Supplementary References:

1 Söderström, B., Chan, H., Shilling, P. J., Skoglund, U. & Daley, D. O. Spatial separation of FtsZ and FtsN during cell division. *Molecular microbiology* **107**, 387-401, doi:10.1111/mmi.13888 (2018).

2 Cabre, E. J. *et al.* Bacterial division proteins FtsZ and ZipA induce vesicle shrinkage and cell membrane invagination. *J Biol Chem* **288**, 26625-26634, doi:10.1074/jbc.M113.491688 (2013).

3 Loose, M. & Mitchison, T. J. The bacterial cell division proteins FtsA and FtsZ self-organize into dynamic cytoskeletal patterns. *Nature cell biology* **16**, 38-46, doi:10.1038/ncb2885 (2014).
