# Supplementary material for: Cell shape-independent FtsZ dynamics in synthetically remodeled bacterial cells
Source: Nat Commun. 2018 Oct 18;9:4323. doi: 10.1038/s41467-018-06887-7 (PMC6193997; doi:10.1038/s41467-018-06887-7)
Supplement: Supplementary file 3 — Description of Additional Supplementary Files [file 41467_2018_6887_MOESM3_ESM.pdf]

Description of Additional Supplementary Files

File Name: Supplementary Movie 1
Description: 3D rendering of a confocal Z-stack of a drug-treated E. coli cell expressing FtsZ-mNeonGreen and trapped in a hole. Only one Z-ring is observed. Scale bar = 1 µm.

File Name: Supplementary Movie 2
Description: Montage of SIM time-lapse images showing FtsZ dynamics in cells expressing FtsZ-GFP, treated with drugs (cephalexin and A22) and trapped in a standing position. The cell in the lower right corner was not treated with drugs. Scale bar = 1 µm.

File Name: Supplementary Movie 3
Description: Representative time-lapse imaging of dynamic ZipA-GFP densities in a drug-exposed cell with large diameter (2.26 µm). Scale bar = 1 µm.

File Name: Supplementary Movie 4
Description: 3D rendering of a confocal Z-stack of a squared-shaped cell expressing FtsZ-mNeonGreen. Scale bar = 1 µm

File Name: Supplementary Movie 5
Description: Montage of two E. coli cells expressing FtsZ-mCitrine, showing typical treadmilling motion in large, square-shaped cells. Scale bar = 1 µm.

File Name: Supplementary Movie 6
Description: Left, FtsZ-GFP treadmilling in a standing, large, squared-shaped E. coli cell. Right, untreated cell expressing FtsZ-GFP, included as a size reference. Scale bar = 1 µm.

File Name: Supplementary Movie 7
Description: FtsZ-GFP treadmilling in a standing, large, squared-shaped E. coli cell. While most FtsZ-GFP filaments were dynamic, a few were not (bright, immobile spot indicated by the red arrow). Scale bar = 1 µm.

File Name: Supplementary Movie 8
Description: FtsZ-mCitrine dynamics in the corner of a rectangular E. coli cell. Scale bar = 1 µm.

File Name: Supplementary Movie 9
Description: Confocal Fluorescence Recovery After Photobleaching (FRAP) measurement on a rectangular E. coli cell expressing FtsZ-GFP. Scale bar = 1 µm.

File Name: Supplementary Movie 10
Description: Montage of time-lapse images of E. coli cells expressing FtsZ-mCitrine and sculptured into various shapes (clock-wise from top left: heart, pentagon, triangle and half-moon). FtsZ-mCitrine appears dynamic in all shapes. A smoothing filter was applied to each movie (Walking Average plug-in in Fiji, average 2). Scale bar = 1 µm.
